# Children’s Health: School Siting Poses Particulate Problem

**DOI:** 10.1289/ehp.116-a474

**Published:** 2008-11

**Authors:** Carol Potera

Numerous studies dating back 18 years indicate that children who live close to major roadways tend to have more respiratory ailments as a result of exposure to nitrogen dioxide, carbon monoxide, ultrafine diesel exhaust particles, and other traffic pollutants. Children spend about 8 hours each weekday in school—and this part of their day may bring no relief from exposure. A new study finds that about one-third of U.S. public schools are located within 400 meters of a major roadway, and about one-tenth are within 100 meters. “School attendance may result in a large dose of inhaled traffic pollutants that have been completely overlooked,” says study leader Sergey Grinshpun, director of the Center for Health-Related Aerosol Studies at the University of Cincinnati in Ohio.

Grinshpun’s team mapped inner-city, suburban, and rural schools located in the Atlanta, Boston, Philadelphia, Cincinnati, Memphis, Denver, Los Angeles, Minneapolis, and San Antonio metropolitan regions. A total of 8,803 public schools attended by 6 million students were included. The distance to the nearest interstate or state highway was estimated using geographic information system software. The team counted how many schools were sited within 400 and 100 meters of a roadway, parameters selected on the basis of previous studies that linked these distances with exposure to traffic pollutants and with chronic respiratory symptoms.

Thirty-three percent of the schools were within 400 meters of a roadway, and 12% were within 100 meters. Schools in the East tended to be closer to roadways than those in the West. Boston, for example, had twice as many schools within 400 meters as Los Angeles (44% versus 20%) and six times more schools within 100 meters (18% versus 3%). Schools in the suburbs were more likely to be closer to busy roadways than inner-city schools, especially in the East. In Boston, Philadelphia, Atlanta, and Cincinnati, more than half the schools located within 400 meters were in the suburbs. The study, the first known national survey of school proximity to roadways and health risks, was reported in the September 2008 issue of the *Journal of Environmental Planning and Management*.

Grinshpun recommends that schools situated near highways be retrofitted with air filtration systems to clean up traffic pollutants, adding that most schools are already equipped with central HVAC systems that can be improved by adding special filters to trap ultrafine diesel exhaust particles. He suggests that outdoor activities such as recess and sports be scheduled to avoid peak rush hour times when traffic pollution levels are highest.

However, George Allen, an environmental engineer at NESCAUM, a nonprofit air management think tank in Boston, points out the need to balance particulate and ozone exposures: “Particulate matter is highest in the morning rush hour and sometimes evening rush hour, [but] ozone peaks at early afternoon in most areas. Both are issues with regard to children’s exposure to air pollution, especially when exercising.”

Grinshpun further recommends that new schools be built at least 400 meters from major roadways. This may not always be feasible, he says, yet policy makers should strive to strike a balance between the economics of urban planning and health concerns when building new schools. In California, a state law already dictates that schools cannot be built within 500 feet (168 meters) of a freeway or other busy traffic corridor. “This is not far enough if you are concerned about traffic air pollution,” Grinshpun says.

Allen sees the 400-meter limit as “difficult to implement,” given that the study defined “major roads” to include state roads, which he points out are often small two-lane roads. “Los Angeles is having trouble siting schools that comply with the law,” he says—the problem is finding land that lies far enough from highways. Conceivably, Allen says, schools may end up being built farther out of town, which could require more students to ride buses (instead of walking or biking) and create more traffic pollution and exposure for children.

“School districts generally are limited to obtaining property that developers don’t want,” points out Michael Hall, marketing director at Fanning/Howey Associates, specialists in educational architecture and engineering, and poor locations often come with poor air quality. But new design approaches can help control exposure to exterior pollutants. For example, displacement ventilation systems push air contaminants toward the ceiling, where they are vented from the room. “These issues are at the forefront of school design,” Hall says.

## Figures and Tables

**Figure:**
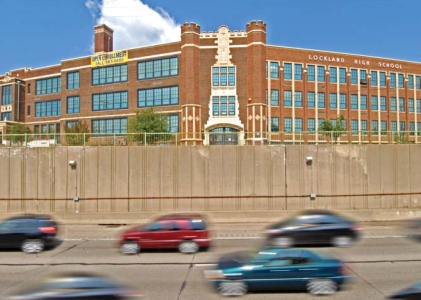
Lockland High School is one of several schools located close to a major highway in Greater Cincinnati.

